# Supplementary material for: Pyruvate Dehydrogenase Kinase 1 inhibition mediated oxidative phosphorylation enhancement in cartilage promotes osteoarthritis progression
Source: BMC Musculoskelet Disord. 2023 Jul 20;24:597. doi: 10.1186/s12891-023-06585-6 (PMC10357736; doi:10.1186/s12891-023-06585-6)
Supplement: Supplementary file 1 — Additional file 1. [file 12891_2023_6585_MOESM1_ESM.zip › MF.Gsea.1653627316242/GOMF_CHROMATIN_BINDING.html]

Details for gene set GOMF\_CHROMATIN\_BINDING[GSEA]

|  || Dataset | 55457\_collapsed\_to\_symbols.cls.cls #OA\_versus\_normal.cls.cls #OA\_versus\_normal\_repos |
| Phenotype | cls.cls#OA\_versus\_normal\_repos |
| Upregulated in class | normal |
| GeneSet | GOMF\_CHROMATIN\_BINDING |
| Enrichment Score (ES) | -0.3068736 |
| Normalized Enrichment Score (NES) | -1.6104223 |
| Nominal p-value | 0.0 |
| FDR q-value | 0.1446139 |
| FWER p-Value | 0.99 |
Table: GSEA Results Summary

  

Fig 1: Enrichment plot: GOMF\_CHROMATIN\_BINDING      
 Profile of the Running ES Score & Positions of GeneSet Members on the Rank Ordered List

  

| SYMBOL | TITLE | RANK IN GENE LIST | RANK METRIC SCORE | RUNNING ES | CORE ENRICHMENT || 1 | ASF1A | anti-silencing function 1A histone chaperone [Source:HGNC Symbol;Acc:HGNC:20995] | 88 | 0.928 | -0.0011 | No |
| 2 | HMGN4 | high mobility group nucleosomal binding domain 4 [Source:HGNC Symbol;Acc:HGNC:4989] | 114 | 0.896 | 0.0026 | No |
| 3 | NDN | "necdin, MAGE family member [Source:HGNC Symbol;Acc:HGNC:7675]" | 191 | 0.827 | 0.0018 | No |
| 4 | NAP1L3 | nucleosome assembly protein 1 like 3 [Source:HGNC Symbol;Acc:HGNC:7639] | 229 | 0.800 | 0.0040 | No |
| 5 | SMAD2 | SMAD family member 2 [Source:HGNC Symbol;Acc:HGNC:6768] | 275 | 0.768 | 0.0053 | No |
| 6 | PAX6 | paired box 6 [Source:HGNC Symbol;Acc:HGNC:8620] | 304 | 0.746 | 0.0079 | No |
| 7 | RAD17 | RAD17 checkpoint clamp loader component [Source:HGNC Symbol;Acc:HGNC:9807] | 306 | 0.744 | 0.0126 | No |
| 8 | HMGN5 | high mobility group nucleosome binding domain 5 [Source:HGNC Symbol;Acc:HGNC:8013] | 376 | 0.716 | 0.0116 | No |
| 9 | SLC30A9 | solute carrier family 30 member 9 [Source:HGNC Symbol;Acc:HGNC:1329] | 390 | 0.710 | 0.0151 | No |
| 10 | DNMT3A | DNA methyltransferase 3 alpha [Source:HGNC Symbol;Acc:HGNC:2978] | 406 | 0.704 | 0.0184 | No |
| 11 | EED | embryonic ectoderm development [Source:HGNC Symbol;Acc:HGNC:3188] | 454 | 0.680 | 0.0190 | No |
| 12 | ZNHIT1 | zinc finger HIT-type containing 1 [Source:HGNC Symbol;Acc:HGNC:21688] | 495 | 0.661 | 0.0201 | No |
| 13 | MLH1 | mutL homolog 1 [Source:HGNC Symbol;Acc:HGNC:7127] | 497 | 0.660 | 0.0242 | No |
| 14 | MEIS1 | Meis homeobox 1 [Source:HGNC Symbol;Acc:HGNC:7000] | 625 | 0.613 | 0.0180 | No |
| 15 | HESX1 | HESX homeobox 1 [Source:HGNC Symbol;Acc:HGNC:4877] | 656 | 0.604 | 0.0194 | No |
| 16 | MSH2 | mutS homolog 2 [Source:HGNC Symbol;Acc:HGNC:7325] | 739 | 0.584 | 0.0166 | No |
| 17 | PARP2 | poly(ADP-ribose) polymerase 2 [Source:HGNC Symbol;Acc:HGNC:272] | 758 | 0.579 | 0.0189 | No |
| 18 | MLLT3 | MLLT3 super elongation complex subunit [Source:HGNC Symbol;Acc:HGNC:7136] | 764 | 0.577 | 0.0222 | No |
| 19 | H1-10 | H1.10 linker histone [Source:HGNC Symbol;Acc:HGNC:4722] | 766 | 0.577 | 0.0258 | No |
| 20 | POLD1 | "DNA polymerase delta 1, catalytic subunit [Source:HGNC Symbol;Acc:HGNC:9175]" | 811 | 0.566 | 0.0259 | No |
| 21 | NPM3 | nucleophosmin/nucleoplasmin 3 [Source:HGNC Symbol;Acc:HGNC:7931] | 868 | 0.551 | 0.0249 | No |
| 22 | NAP1L2 | nucleosome assembly protein 1 like 2 [Source:HGNC Symbol;Acc:HGNC:7638] | 935 | 0.531 | 0.0230 | No |
| 23 | MBD5 | methyl-CpG binding domain protein 5 [Source:HGNC Symbol;Acc:HGNC:20444] | 941 | 0.530 | 0.0260 | No |
| 24 | HPF1 | histone PARylation factor 1 [Source:HGNC Symbol;Acc:HGNC:26051] | 965 | 0.525 | 0.0276 | No |
| 25 | ESR1 | estrogen receptor 1 [Source:HGNC Symbol;Acc:HGNC:3467] | 984 | 0.522 | 0.0295 | No |
| 26 | NEUROD1 | neuronal differentiation 1 [Source:HGNC Symbol;Acc:HGNC:7762] | 1008 | 0.515 | 0.0309 | No |
| 27 | NELFE | negative elongation factor complex member E [Source:HGNC Symbol;Acc:HGNC:13974] | 1135 | 0.489 | 0.0239 | No |
| 28 | KDM4D | lysine demethylase 4D [Source:HGNC Symbol;Acc:HGNC:25498] | 1180 | 0.480 | 0.0235 | No |
| 29 | RBBP4 | "RB binding protein 4, chromatin remodeling factor [Source:HGNC Symbol;Acc:HGNC:9887]" | 1181 | 0.480 | 0.0266 | No |
| 30 | SMC2 | structural maintenance of chromosomes 2 [Source:HGNC Symbol;Acc:HGNC:14011] | 1190 | 0.479 | 0.0290 | No |
| 31 | GTF2B | general transcription factor IIB [Source:HGNC Symbol;Acc:HGNC:4648] | 1315 | 0.455 | 0.0219 | No |
| 32 | URI1 | URI1 prefoldin like chaperone [Source:HGNC Symbol;Acc:HGNC:13236] | 1324 | 0.454 | 0.0242 | No |
| 33 | HDAC3 | histone deacetylase 3 [Source:HGNC Symbol;Acc:HGNC:4854] | 1325 | 0.454 | 0.0271 | No |
| 34 | CBX1 | chromobox 1 [Source:HGNC Symbol;Acc:HGNC:1551] | 1331 | 0.453 | 0.0296 | No |
| 35 | TSPYL5 | TSPY like 5 [Source:HGNC Symbol;Acc:HGNC:29367] | 1338 | 0.452 | 0.0321 | No |
| 36 | WBP2 | WW domain binding protein 2 [Source:HGNC Symbol;Acc:HGNC:12738] | 1344 | 0.451 | 0.0346 | No |
| 37 | HMGN1 | high mobility group nucleosome binding domain 1 [Source:HGNC Symbol;Acc:HGNC:4984] | 1362 | 0.448 | 0.0361 | No |
| 38 | CBX4 | chromobox 4 [Source:HGNC Symbol;Acc:HGNC:1554] | 1397 | 0.443 | 0.0362 | No |
| 39 | ADNP | activity dependent neuroprotector homeobox [Source:HGNC Symbol;Acc:HGNC:15766] | 1466 | 0.434 | 0.0335 | No |
| 40 | BCAS3 | BCAS3 microtubule associated cell migration factor [Source:HGNC Symbol;Acc:HGNC:14347] | 1471 | 0.433 | 0.0360 | No |
| 41 | SHMT2 | serine hydroxymethyltransferase 2 [Source:HGNC Symbol;Acc:HGNC:10852] | 1518 | 0.426 | 0.0350 | No |
| 42 | PCNA | proliferating cell nuclear antigen [Source:HGNC Symbol;Acc:HGNC:8729] | 1557 | 0.419 | 0.0346 | No |
| 43 | RAD51 | RAD51 recombinase [Source:HGNC Symbol;Acc:HGNC:9817] | 1581 | 0.416 | 0.0355 | No |
| 44 | UXT | ubiquitously expressed prefoldin like chaperone [Source:HGNC Symbol;Acc:HGNC:12641] | 1641 | 0.407 | 0.0333 | No |
| 45 | GPER1 | G protein-coupled estrogen receptor 1 [Source:HGNC Symbol;Acc:HGNC:4485] | 1677 | 0.402 | 0.0331 | No |
| 46 | ERCC4 | "ERCC excision repair 4, endonuclease catalytic subunit [Source:HGNC Symbol;Acc:HGNC:3436]" | 1696 | 0.400 | 0.0342 | No |
| 47 | TSPYL4 | TSPY like 4 [Source:HGNC Symbol;Acc:HGNC:21559] | 1713 | 0.396 | 0.0355 | No |
| 48 | NUDT21 | nudix hydrolase 21 [Source:HGNC Symbol;Acc:HGNC:13870] | 1734 | 0.393 | 0.0364 | No |
| 49 | ZNF304 | zinc finger protein 304 [Source:HGNC Symbol;Acc:HGNC:13505] | 1739 | 0.393 | 0.0386 | No |
| 50 | CEBPA | CCAAT enhancer binding protein alpha [Source:HGNC Symbol;Acc:HGNC:1833] | 1750 | 0.392 | 0.0403 | No |
| 51 | STAG3L4 | stromal antigen 3-like 4 (pseudogene) [Source:HGNC Symbol;Acc:HGNC:33887] | 1759 | 0.389 | 0.0422 | No |
| 52 | CSNK2B | casein kinase 2 beta [Source:HGNC Symbol;Acc:HGNC:2460] | 1788 | 0.385 | 0.0424 | No |
| 53 | TOX | thymocyte selection associated high mobility group box [Source:HGNC Symbol;Acc:HGNC:18988] | 1927 | 0.369 | 0.0337 | No |
| 54 | MBD2 | methyl-CpG binding domain protein 2 [Source:HGNC Symbol;Acc:HGNC:6917] | 1958 | 0.366 | 0.0336 | No |
| 55 | SIX1 | SIX homeobox 1 [Source:HGNC Symbol;Acc:HGNC:10887] | 2011 | 0.360 | 0.0317 | No |
| 56 | SOX9 | SRY-box transcription factor 9 [Source:HGNC Symbol;Acc:HGNC:11204] | 2089 | 0.351 | 0.0278 | No |
| 57 | FLI1 | "Fli-1 proto-oncogene, ETS transcription factor [Source:HGNC Symbol;Acc:HGNC:3749]" | 2092 | 0.350 | 0.0299 | No |
| 58 | GRHL2 | grainyhead like transcription factor 2 [Source:HGNC Symbol;Acc:HGNC:2799] | 2111 | 0.348 | 0.0307 | No |
| 59 | CTCF | CCCTC-binding factor [Source:HGNC Symbol;Acc:HGNC:13723] | 2183 | 0.340 | 0.0271 | No |
| 60 | RIT2 | Ras like without CAAX 2 [Source:HGNC Symbol;Acc:HGNC:10017] | 2206 | 0.338 | 0.0275 | No |
| 61 | PCGF1 | polycomb group ring finger 1 [Source:HGNC Symbol;Acc:HGNC:17615] | 2272 | 0.329 | 0.0244 | No |
| 62 | FEZF2 | FEZ family zinc finger 2 [Source:HGNC Symbol;Acc:HGNC:13506] | 2278 | 0.329 | 0.0261 | No |
| 63 | POLA1 | "DNA polymerase alpha 1, catalytic subunit [Source:HGNC Symbol;Acc:HGNC:9173]" | 2300 | 0.327 | 0.0265 | No |
| 64 | ISL1 | ISL LIM homeobox 1 [Source:HGNC Symbol;Acc:HGNC:6132] | 2373 | 0.318 | 0.0228 | No |
| 65 | MSL1 | MSL complex subunit 1 [Source:HGNC Symbol;Acc:HGNC:27905] | 2392 | 0.315 | 0.0234 | No |
| 66 | ZNF750 | zinc finger protein 750 [Source:HGNC Symbol;Acc:HGNC:25843] | 2449 | 0.308 | 0.0208 | No |
| 67 | PCGF2 | polycomb group ring finger 2 [Source:HGNC Symbol;Acc:HGNC:12929] | 2452 | 0.308 | 0.0227 | No |
| 68 | AUTS2 | activator of transcription and developmental regulator AUTS2 [Source:HGNC Symbol;Acc:HGNC:14262] | 2504 | 0.302 | 0.0205 | No |
| 69 | H1-5 | "H1.5 linker histone, cluster member [Source:HGNC Symbol;Acc:HGNC:4719]" | 2518 | 0.301 | 0.0214 | No |
| 70 | ORC1 | origin recognition complex subunit 1 [Source:HGNC Symbol;Acc:HGNC:8487] | 2525 | 0.300 | 0.0228 | No |
| 71 | INSM1 | INSM transcriptional repressor 1 [Source:HGNC Symbol;Acc:HGNC:6090] | 2531 | 0.299 | 0.0244 | No |
| 72 | PKN1 | protein kinase N1 [Source:HGNC Symbol;Acc:HGNC:9405] | 2540 | 0.298 | 0.0256 | No |
| 73 | MED12 | mediator complex subunit 12 [Source:HGNC Symbol;Acc:HGNC:11957] | 2588 | 0.292 | 0.0237 | No |
| 74 | KDM8 | lysine demethylase 8 [Source:HGNC Symbol;Acc:HGNC:25840] | 2594 | 0.291 | 0.0252 | No |
| 75 | APTX | aprataxin [Source:HGNC Symbol;Acc:HGNC:15984] | 2702 | 0.279 | 0.0184 | No |
| 76 | SOX15 | SRY-box transcription factor 15 [Source:HGNC Symbol;Acc:HGNC:11196] | 2748 | 0.275 | 0.0165 | No |
| 77 | H2AZ1 | H2A.Z variant histone 1 [Source:HGNC Symbol;Acc:HGNC:4741] | 2756 | 0.275 | 0.0177 | No |
| 78 | STAG3 | stromal antigen 3 [Source:HGNC Symbol;Acc:HGNC:11356] | 2761 | 0.274 | 0.0192 | No |
| 79 | RUVBL2 | RuvB like AAA ATPase 2 [Source:HGNC Symbol;Acc:HGNC:10475] | 2778 | 0.272 | 0.0196 | No |
| 80 | DNMT1 | DNA methyltransferase 1 [Source:HGNC Symbol;Acc:HGNC:2976] | 2836 | 0.267 | 0.0168 | No |
| 81 | GLI3 | GLI family zinc finger 3 [Source:HGNC Symbol;Acc:HGNC:4319] | 2861 | 0.264 | 0.0165 | No |
| 82 | PCLAF | PCNA clamp associated factor [Source:HGNC Symbol;Acc:HGNC:28961] | 2889 | 0.262 | 0.0160 | No |
| 83 | H1-0 | H1.0 linker histone [Source:HGNC Symbol;Acc:HGNC:4714] | 2931 | 0.258 | 0.0144 | No |
| 84 | ERCC1 | "ERCC excision repair 1, endonuclease non-catalytic subunit [Source:HGNC Symbol;Acc:HGNC:3433]" | 2948 | 0.255 | 0.0148 | No |
| 85 | VAX2 | ventral anterior homeobox 2 [Source:HGNC Symbol;Acc:HGNC:12661] | 2950 | 0.255 | 0.0163 | No |
| 86 | SSBP1 | single stranded DNA binding protein 1 [Source:HGNC Symbol;Acc:HGNC:11317] | 3038 | 0.246 | 0.0109 | No |
| 87 | PLAC8 | placenta associated 8 [Source:HGNC Symbol;Acc:HGNC:19254] | 3093 | 0.240 | 0.0081 | No |
| 88 | DPPA4 | developmental pluripotency associated 4 [Source:HGNC Symbol;Acc:HGNC:19200] | 3106 | 0.239 | 0.0087 | No |
| 89 | CENPS | centromere protein S [Source:HGNC Symbol;Acc:HGNC:23163] | 3184 | 0.232 | 0.0040 | No |
| 90 | CLOCK | clock circadian regulator [Source:HGNC Symbol;Acc:HGNC:2082] | 3231 | 0.227 | 0.0017 | No |
| 91 | SMARCA2 | "SWI/SNF related, matrix associated, actin dependent regulator of chromatin, subfamily a, member 2 [Source:HGNC Symbol;Acc:HGNC:11098]" | 3281 | 0.222 | -0.0008 | No |
| 92 | DDX1 | DEAD-box helicase 1 [Source:HGNC Symbol;Acc:HGNC:2734] | 3305 | 0.221 | -0.0012 | No |
| 93 | ACTR6 | actin related protein 6 [Source:HGNC Symbol;Acc:HGNC:24025] | 3317 | 0.219 | -0.0007 | No |
| 94 | HR | HR lysine demethylase and nuclear receptor corepressor [Source:HGNC Symbol;Acc:HGNC:5172] | 3356 | 0.216 | -0.0024 | No |
| 95 | ELK1 | ETS transcription factor ELK1 [Source:HGNC Symbol;Acc:HGNC:3321] | 3357 | 0.216 | -0.0010 | No |
| 96 | TOX3 | TOX high mobility group box family member 3 [Source:HGNC Symbol;Acc:HGNC:11972] | 3438 | 0.207 | -0.0061 | No |
| 97 | ONECUT1 | one cut homeobox 1 [Source:HGNC Symbol;Acc:HGNC:8138] | 3443 | 0.207 | -0.0051 | No |
| 98 | TOP2B | DNA topoisomerase II beta [Source:HGNC Symbol;Acc:HGNC:11990] | 3458 | 0.206 | -0.0049 | No |
| 99 | SMAD6 | SMAD family member 6 [Source:HGNC Symbol;Acc:HGNC:6772] | 3460 | 0.206 | -0.0036 | No |
| 100 | TRIM28 | tripartite motif containing 28 [Source:HGNC Symbol;Acc:HGNC:16384] | 3526 | 0.200 | -0.0076 | No |
| 101 | ENY2 | ENY2 transcription and export complex 2 subunit [Source:HGNC Symbol;Acc:HGNC:24449] | 3561 | 0.197 | -0.0091 | No |
| 102 | EZH1 | enhancer of zeste 1 polycomb repressive complex 2 subunit [Source:HGNC Symbol;Acc:HGNC:3526] | 3565 | 0.197 | -0.0080 | No |
| 103 | SMC4 | structural maintenance of chromosomes 4 [Source:HGNC Symbol;Acc:HGNC:14013] | 3574 | 0.196 | -0.0074 | No |
| 104 | ATAD2 | ATPase family AAA domain containing 2 [Source:HGNC Symbol;Acc:HGNC:30123] | 3597 | 0.194 | -0.0079 | No |
| 105 | WDHD1 | WD repeat and HMG-box DNA binding protein 1 [Source:HGNC Symbol;Acc:HGNC:23170] | 3690 | 0.186 | -0.0141 | No |
| 106 | CDK1 | cyclin dependent kinase 1 [Source:HGNC Symbol;Acc:HGNC:1722] | 3744 | 0.182 | -0.0172 | No |
| 107 | MACROH2A2 | macroH2A.2 histone [Source:HGNC Symbol;Acc:HGNC:14453] | 3793 | 0.178 | -0.0200 | No |
| 108 | HMGN3 | high mobility group nucleosomal binding domain 3 [Source:HGNC Symbol;Acc:HGNC:12312] | 3818 | 0.174 | -0.0208 | No |
| 109 | RNF4 | ring finger protein 4 [Source:HGNC Symbol;Acc:HGNC:10067] | 3851 | 0.170 | -0.0223 | No |
| 110 | POLE3 | "DNA polymerase epsilon 3, accessory subunit [Source:HGNC Symbol;Acc:HGNC:13546]" | 3934 | 0.162 | -0.0278 | No |
| 111 | RELA | "RELA proto-oncogene, NF-kB subunit [Source:HGNC Symbol;Acc:HGNC:9955]" | 3942 | 0.161 | -0.0273 | No |
| 112 | LEMD3 | LEM domain containing 3 [Source:HGNC Symbol;Acc:HGNC:28887] | 3947 | 0.161 | -0.0266 | No |
| 113 | MORF4L1 | mortality factor 4 like 1 [Source:HGNC Symbol;Acc:HGNC:16989] | 3984 | 0.157 | -0.0285 | No |
| 114 | RUNX2 | RUNX family transcription factor 2 [Source:HGNC Symbol;Acc:HGNC:10472] | 3993 | 0.157 | -0.0282 | No |
| 115 | SMAD3 | SMAD family member 3 [Source:HGNC Symbol;Acc:HGNC:6769] | 4061 | 0.150 | -0.0326 | No |
| 116 | GADD45A | growth arrest and DNA damage inducible alpha [Source:HGNC Symbol;Acc:HGNC:4095] | 4081 | 0.148 | -0.0332 | No |
| 117 | SMARCE1 | "SWI/SNF related, matrix associated, actin dependent regulator of chromatin, subfamily e, member 1 [Source:HGNC Symbol;Acc:HGNC:11109]" | 4255 | 0.132 | -0.0462 | No |
| 118 | NCOA2 | nuclear receptor coactivator 2 [Source:HGNC Symbol;Acc:HGNC:7669] | 4284 | 0.129 | -0.0477 | No |
| 119 | POLQ | DNA polymerase theta [Source:HGNC Symbol;Acc:HGNC:9186] | 4293 | 0.129 | -0.0475 | No |
| 120 | MLH3 | mutL homolog 3 [Source:HGNC Symbol;Acc:HGNC:7128] | 4335 | 0.125 | -0.0500 | No |
| 121 | ATOH1 | atonal bHLH transcription factor 1 [Source:HGNC Symbol;Acc:HGNC:797] | 4428 | 0.119 | -0.0566 | No |
| 122 | UPF1 | UPF1 RNA helicase and ATPase [Source:HGNC Symbol;Acc:HGNC:9962] | 4434 | 0.118 | -0.0563 | No |
| 123 | CENPF | centromere protein F [Source:HGNC Symbol;Acc:HGNC:1857] | 4437 | 0.118 | -0.0557 | No |
| 124 | NEUROG3 | neurogenin 3 [Source:HGNC Symbol;Acc:HGNC:13806] | 4443 | 0.118 | -0.0553 | No |
| 125 | IFT74 | intraflagellar transport 74 [Source:HGNC Symbol;Acc:HGNC:21424] | 4470 | 0.115 | -0.0566 | No |
| 126 | STAT1 | signal transducer and activator of transcription 1 [Source:HGNC Symbol;Acc:HGNC:11362] | 4495 | 0.113 | -0.0579 | No |
| 127 | HMGN2 | high mobility group nucleosomal binding domain 2 [Source:HGNC Symbol;Acc:HGNC:4986] | 4528 | 0.111 | -0.0597 | No |
| 128 | KLHDC3 | kelch domain containing 3 [Source:HGNC Symbol;Acc:HGNC:20704] | 4598 | 0.105 | -0.0646 | No |
| 129 | NFKB1 | nuclear factor kappa B subunit 1 [Source:HGNC Symbol;Acc:HGNC:7794] | 4626 | 0.102 | -0.0661 | No |
| 130 | ASXL2 | ASXL transcriptional regulator 2 [Source:HGNC Symbol;Acc:HGNC:23805] | 4639 | 0.101 | -0.0664 | No |
| 131 | STAG2 | stromal antigen 2 [Source:HGNC Symbol;Acc:HGNC:11355] | 4684 | 0.098 | -0.0693 | No |
| 132 | SREBF1 | sterol regulatory element binding transcription factor 1 [Source:HGNC Symbol;Acc:HGNC:11289] | 4729 | 0.093 | -0.0723 | No |
| 133 | MITF | melanocyte inducing transcription factor [Source:HGNC Symbol;Acc:HGNC:7105] | 4759 | 0.090 | -0.0740 | No |
| 134 | TSPYL1 | TSPY like 1 [Source:HGNC Symbol;Acc:HGNC:12382] | 4829 | 0.085 | -0.0790 | No |
| 135 | HNRNPD | heterogeneous nuclear ribonucleoprotein D [Source:HGNC Symbol;Acc:HGNC:5036] | 4830 | 0.085 | -0.0785 | No |
| 136 | HOXD13 | homeobox D13 [Source:HGNC Symbol;Acc:HGNC:5136] | 4837 | 0.084 | -0.0784 | No |
| 137 | KAT2B | lysine acetyltransferase 2B [Source:HGNC Symbol;Acc:HGNC:8638] | 4867 | 0.082 | -0.0802 | No |
| 138 | SETDB1 | SET domain bifurcated histone lysine methyltransferase 1 [Source:HGNC Symbol;Acc:HGNC:10761] | 4891 | 0.080 | -0.0816 | No |
| 139 | HCFC1 | host cell factor C1 [Source:HGNC Symbol;Acc:HGNC:4839] | 4937 | 0.077 | -0.0847 | No |
| 140 | RNF8 | ring finger protein 8 [Source:HGNC Symbol;Acc:HGNC:10071] | 4938 | 0.077 | -0.0842 | No |
| 141 | SMC1A | structural maintenance of chromosomes 1A [Source:HGNC Symbol;Acc:HGNC:11111] | 4942 | 0.077 | -0.0840 | No |
| 142 | CBX5 | chromobox 5 [Source:HGNC Symbol;Acc:HGNC:1555] | 4963 | 0.075 | -0.0851 | No |
| 143 | CDYL | chromodomain Y like [Source:HGNC Symbol;Acc:HGNC:1811] | 5016 | 0.072 | -0.0888 | No |
| 144 | OVOL2 | ovo like zinc finger 2 [Source:HGNC Symbol;Acc:HGNC:15804] | 5017 | 0.071 | -0.0884 | No |
| 145 | STAG1 | stromal antigen 1 [Source:HGNC Symbol;Acc:HGNC:11354] | 5022 | 0.071 | -0.0882 | No |
| 146 | YAP1 | Yes1 associated transcriptional regulator [Source:HGNC Symbol;Acc:HGNC:16262] | 5041 | 0.070 | -0.0892 | No |
| 147 | CTBP2 | C-terminal binding protein 2 [Source:HGNC Symbol;Acc:HGNC:2495] | 5066 | 0.067 | -0.0907 | No |
| 148 | KDM6A | lysine demethylase 6A [Source:HGNC Symbol;Acc:HGNC:12637] | 5069 | 0.067 | -0.0904 | No |
| 149 | SAP30L | SAP30 like [Source:HGNC Symbol;Acc:HGNC:25663] | 5124 | 0.062 | -0.0944 | No |
| 150 | SMARCD2 | "SWI/SNF related, matrix associated, actin dependent regulator of chromatin, subfamily d, member 2 [Source:HGNC Symbol;Acc:HGNC:11107]" | 5129 | 0.062 | -0.0943 | No |
| 151 | SUPT16H | "SPT16 homolog, facilitates chromatin remodeling subunit [Source:HGNC Symbol;Acc:HGNC:11465]" | 5174 | 0.058 | -0.0975 | No |
| 152 | NSD1 | nuclear receptor binding SET domain protein 1 [Source:HGNC Symbol;Acc:HGNC:14234] | 5178 | 0.058 | -0.0974 | No |
| 153 | ZNF274 | zinc finger protein 274 [Source:HGNC Symbol;Acc:HGNC:13068] | 5181 | 0.058 | -0.0971 | No |
| 154 | TSPY1 | testis specific protein Y-linked 1 [Source:HGNC Symbol;Acc:HGNC:12381] | 5214 | 0.055 | -0.0994 | No |
| 155 | FOXC1 | forkhead box C1 [Source:HGNC Symbol;Acc:HGNC:3800] | 5227 | 0.054 | -0.1000 | No |
| 156 | TFAP2A | transcription factor AP-2 alpha [Source:HGNC Symbol;Acc:HGNC:11742] | 5379 | 0.041 | -0.1119 | No |
| 157 | MEOX1 | mesenchyme homeobox 1 [Source:HGNC Symbol;Acc:HGNC:7013] | 5384 | 0.041 | -0.1119 | No |
| 158 | TAL1 | "TAL bHLH transcription factor 1, erythroid differentiation factor [Source:HGNC Symbol;Acc:HGNC:11556]" | 5406 | 0.040 | -0.1134 | No |
| 159 | H1-1 | "H1.1 linker histone, cluster member [Source:HGNC Symbol;Acc:HGNC:4715]" | 5447 | 0.037 | -0.1163 | No |
| 160 | EHMT2 | euchromatic histone lysine methyltransferase 2 [Source:HGNC Symbol;Acc:HGNC:14129] | 5507 | 0.032 | -0.1209 | No |
| 161 | TP53 | tumor protein p53 [Source:HGNC Symbol;Acc:HGNC:11998] | 5511 | 0.032 | -0.1209 | No |
| 162 | LHX2 | LIM homeobox 2 [Source:HGNC Symbol;Acc:HGNC:6594] | 5516 | 0.031 | -0.1210 | No |
| 163 | GATA6 | GATA binding protein 6 [Source:HGNC Symbol;Acc:HGNC:4174] | 5537 | 0.030 | -0.1225 | No |
| 164 | STAT5B | signal transducer and activator of transcription 5B [Source:HGNC Symbol;Acc:HGNC:11367] | 5554 | 0.028 | -0.1236 | No |
| 165 | NUPR1 | "nuclear protein 1, transcriptional regulator [Source:HGNC Symbol;Acc:HGNC:29990]" | 5616 | 0.023 | -0.1283 | No |
| 166 | MEF2A | myocyte enhancer factor 2A [Source:HGNC Symbol;Acc:HGNC:6993] | 5628 | 0.022 | -0.1291 | No |
| 167 | THRA | thyroid hormone receptor alpha [Source:HGNC Symbol;Acc:HGNC:11796] | 5638 | 0.021 | -0.1297 | No |
| 168 | SMARCA1 | "SWI/SNF related, matrix associated, actin dependent regulator of chromatin, subfamily a, member 1 [Source:HGNC Symbol;Acc:HGNC:11097]" | 5723 | 0.014 | -0.1363 | No |
| 169 | ARID1A | AT-rich interaction domain 1A [Source:HGNC Symbol;Acc:HGNC:11110] | 5735 | 0.013 | -0.1371 | No |
| 170 | OBI1 | ORC ubiquitin ligase 1 [Source:HGNC Symbol;Acc:HGNC:20308] | 5736 | 0.012 | -0.1371 | No |
| 171 | MBD3 | methyl-CpG binding domain protein 3 [Source:HGNC Symbol;Acc:HGNC:6918] | 5795 | 0.008 | -0.1417 | No |
| 172 | PRDM11 | PR/SET domain 11 [Source:HGNC Symbol;Acc:HGNC:13996] | 5876 | 0.004 | -0.1481 | No |
| 173 | BRIP1 | BRCA1 interacting helicase 1 [Source:HGNC Symbol;Acc:HGNC:20473] | 5885 | 0.003 | -0.1487 | No |
| 174 | PAF1 | "PAF1 homolog, Paf1/RNA polymerase II complex component [Source:HGNC Symbol;Acc:HGNC:25459]" | 5901 | 0.002 | -0.1499 | No |
| 175 | TPR | "translocated promoter region, nuclear basket protein [Source:HGNC Symbol;Acc:HGNC:12017]" | 5919 | 0.001 | -0.1513 | No |
| 176 | MACROH2A1 | macroH2A.1 histone [Source:HGNC Symbol;Acc:HGNC:4740] | 5953 | -0.002 | -0.1539 | No |
| 177 | MTF2 | metal response element binding transcription factor 2 [Source:HGNC Symbol;Acc:HGNC:29535] | 5996 | -0.004 | -0.1573 | No |
| 178 | KDM3B | lysine demethylase 3B [Source:HGNC Symbol;Acc:HGNC:1337] | 6005 | -0.005 | -0.1579 | No |
| 179 | PPARG | peroxisome proliferator activated receptor gamma [Source:HGNC Symbol;Acc:HGNC:9236] | 6006 | -0.005 | -0.1579 | No |
| 180 | SUV39H1 | suppressor of variegation 3-9 homolog 1 [Source:HGNC Symbol;Acc:HGNC:11479] | 6028 | -0.007 | -0.1595 | No |
| 181 | ARID3A | AT-rich interaction domain 3A [Source:HGNC Symbol;Acc:HGNC:3031] | 6031 | -0.007 | -0.1596 | No |
| 182 | ING2 | inhibitor of growth family member 2 [Source:HGNC Symbol;Acc:HGNC:6063] | 6150 | -0.016 | -0.1690 | No |
| 183 | TRIM37 | tripartite motif containing 37 [Source:HGNC Symbol;Acc:HGNC:7523] | 6176 | -0.019 | -0.1709 | No |
| 184 | KMT5B | lysine methyltransferase 5B [Source:HGNC Symbol;Acc:HGNC:24283] | 6198 | -0.020 | -0.1725 | No |
| 185 | TP63 | tumor protein p63 [Source:HGNC Symbol;Acc:HGNC:15979] | 6274 | -0.025 | -0.1783 | No |
| 186 | SIRT2 | sirtuin 2 [Source:HGNC Symbol;Acc:HGNC:10886] | 6279 | -0.026 | -0.1785 | No |
| 187 | NAP1L4 | nucleosome assembly protein 1 like 4 [Source:HGNC Symbol;Acc:HGNC:7640] | 6289 | -0.027 | -0.1790 | No |
| 188 | SCMH1 | Scm polycomb group protein homolog 1 [Source:HGNC Symbol;Acc:HGNC:19003] | 6363 | -0.032 | -0.1847 | No |
| 189 | TOP2A | DNA topoisomerase II alpha [Source:HGNC Symbol;Acc:HGNC:11989] | 6377 | -0.034 | -0.1855 | No |
| 190 | POLR2B | RNA polymerase II subunit B [Source:HGNC Symbol;Acc:HGNC:9188] | 6426 | -0.037 | -0.1892 | No |
| 191 | CDT1 | chromatin licensing and DNA replication factor 1 [Source:HGNC Symbol;Acc:HGNC:24576] | 6448 | -0.039 | -0.1906 | No |
| 192 | PRKCB | protein kinase C beta [Source:HGNC Symbol;Acc:HGNC:9395] | 6453 | -0.039 | -0.1907 | No |
| 193 | CABIN1 | calcineurin binding protein 1 [Source:HGNC Symbol;Acc:HGNC:24187] | 6477 | -0.040 | -0.1923 | No |
| 194 | H1-4 | "H1.4 linker histone, cluster member [Source:HGNC Symbol;Acc:HGNC:4718]" | 6487 | -0.041 | -0.1927 | No |
| 195 | NCOR2 | nuclear receptor corepressor 2 [Source:HGNC Symbol;Acc:HGNC:7673] | 6497 | -0.042 | -0.1932 | No |
| 196 | FOXO1 | forkhead box O1 [Source:HGNC Symbol;Acc:HGNC:3819] | 6511 | -0.043 | -0.1940 | No |
| 197 | HELLS | "helicase, lymphoid specific [Source:HGNC Symbol;Acc:HGNC:4861]" | 6534 | -0.045 | -0.1954 | No |
| 198 | EZH2 | enhancer of zeste 2 polycomb repressive complex 2 subunit [Source:HGNC Symbol;Acc:HGNC:3527] | 6562 | -0.047 | -0.1973 | No |
| 199 | RBL2 | RB transcriptional corepressor like 2 [Source:HGNC Symbol;Acc:HGNC:9894] | 6764 | -0.060 | -0.2131 | No |
| 200 | HINFP | histone H4 transcription factor [Source:HGNC Symbol;Acc:HGNC:17850] | 6776 | -0.061 | -0.2136 | No |
| 201 | NUP62 | nucleoporin 62 [Source:HGNC Symbol;Acc:HGNC:8066] | 6800 | -0.063 | -0.2150 | No |
| 202 | L3MBTL1 | L3MBTL histone methyl-lysine binding protein 1 [Source:HGNC Symbol;Acc:HGNC:15905] | 6807 | -0.063 | -0.2151 | No |
| 203 | HMGA2 | high mobility group AT-hook 2 [Source:HGNC Symbol;Acc:HGNC:5009] | 6850 | -0.067 | -0.2181 | No |
| 204 | ATAD2B | ATPase family AAA domain containing 2B [Source:HGNC Symbol;Acc:HGNC:29230] | 6896 | -0.072 | -0.2212 | No |
| 205 | IKZF3 | IKAROS family zinc finger 3 [Source:HGNC Symbol;Acc:HGNC:13178] | 6905 | -0.073 | -0.2214 | No |
| 206 | MRNIP | MRN complex interacting protein [Source:HGNC Symbol;Acc:HGNC:30817] | 6939 | -0.075 | -0.2236 | No |
| 207 | SMC3 | structural maintenance of chromosomes 3 [Source:HGNC Symbol;Acc:HGNC:2468] | 6950 | -0.076 | -0.2239 | No |
| 208 | ASXL3 | ASXL transcriptional regulator 3 [Source:HGNC Symbol;Acc:HGNC:29357] | 6953 | -0.076 | -0.2236 | No |
| 209 | CENPB | centromere protein B [Source:HGNC Symbol;Acc:HGNC:1852] | 6977 | -0.078 | -0.2249 | No |
| 210 | JMJD1C | jumonji domain containing 1C [Source:HGNC Symbol;Acc:HGNC:12313] | 6989 | -0.079 | -0.2253 | No |
| 211 | SP3 | Sp3 transcription factor [Source:HGNC Symbol;Acc:HGNC:11208] | 7033 | -0.082 | -0.2282 | No |
| 212 | CITED2 | Cbp/p300 interacting transactivator with Glu/Asp rich carboxy-terminal domain 2 [Source:HGNC Symbol;Acc:HGNC:1987] | 7100 | -0.087 | -0.2330 | No |
| 213 | POLR2A | RNA polymerase II subunit A [Source:HGNC Symbol;Acc:HGNC:9187] | 7103 | -0.088 | -0.2326 | No |
| 214 | HNF4A | hepatocyte nuclear factor 4 alpha [Source:HGNC Symbol;Acc:HGNC:5024] | 7160 | -0.093 | -0.2365 | No |
| 215 | NCOA6 | nuclear receptor coactivator 6 [Source:HGNC Symbol;Acc:HGNC:15936] | 7171 | -0.093 | -0.2367 | No |
| 216 | PKNOX1 | PBX/knotted 1 homeobox 1 [Source:HGNC Symbol;Acc:HGNC:9022] | 7225 | -0.097 | -0.2403 | No |
| 217 | NCAPH | non-SMC condensin I complex subunit H [Source:HGNC Symbol;Acc:HGNC:1112] | 7257 | -0.100 | -0.2422 | No |
| 218 | CREB3L1 | cAMP responsive element binding protein 3 like 1 [Source:HGNC Symbol;Acc:HGNC:18856] | 7324 | -0.104 | -0.2468 | No |
| 219 | SOX14 | SRY-box transcription factor 14 [Source:HGNC Symbol;Acc:HGNC:11193] | 7327 | -0.104 | -0.2463 | No |
| 220 | H3-3B | H3.3 histone B [Source:HGNC Symbol;Acc:HGNC:4765] | 7351 | -0.106 | -0.2475 | No |
| 221 | ASCL1 | achaete-scute family bHLH transcription factor 1 [Source:HGNC Symbol;Acc:HGNC:738] | 7375 | -0.108 | -0.2486 | No |
| 222 | ANKRD17 | ankyrin repeat domain 17 [Source:HGNC Symbol;Acc:HGNC:23575] | 7385 | -0.109 | -0.2487 | No |
| 223 | MPHOSPH8 | M-phase phosphoprotein 8 [Source:HGNC Symbol;Acc:HGNC:29810] | 7413 | -0.112 | -0.2501 | No |
| 224 | PHF1 | PHD finger protein 1 [Source:HGNC Symbol;Acc:HGNC:8919] | 7429 | -0.113 | -0.2506 | No |
| 225 | HNRNPC | heterogeneous nuclear ribonucleoprotein C [Source:HGNC Symbol;Acc:HGNC:5035] | 7437 | -0.114 | -0.2504 | No |
| 226 | CHAF1B | chromatin assembly factor 1 subunit B [Source:HGNC Symbol;Acc:HGNC:1911] | 7461 | -0.115 | -0.2515 | No |
| 227 | RAG2 | recombination activating 2 [Source:HGNC Symbol;Acc:HGNC:9832] | 7462 | -0.115 | -0.2508 | No |
| 228 | LEF1 | lymphoid enhancer binding factor 1 [Source:HGNC Symbol;Acc:HGNC:6551] | 7467 | -0.116 | -0.2504 | No |
| 229 | DMRT1 | doublesex and mab-3 related transcription factor 1 [Source:HGNC Symbol;Acc:HGNC:2934] | 7508 | -0.119 | -0.2528 | No |
| 230 | MEN1 | menin 1 [Source:HGNC Symbol;Acc:HGNC:7010] | 7542 | -0.122 | -0.2547 | No |
| 231 | ATRX | ATRX chromatin remodeler [Source:HGNC Symbol;Acc:HGNC:886] | 7551 | -0.123 | -0.2545 | No |
| 232 | CALCOCO1 | calcium binding and coiled-coil domain 1 [Source:HGNC Symbol;Acc:HGNC:29306] | 7559 | -0.124 | -0.2543 | No |
| 233 | FABP1 | fatty acid binding protein 1 [Source:HGNC Symbol;Acc:HGNC:3555] | 7604 | -0.127 | -0.2570 | No |
| 234 | NSD2 | nuclear receptor binding SET domain protein 2 [Source:HGNC Symbol;Acc:HGNC:12766] | 7622 | -0.129 | -0.2576 | No |
| 235 | WDR13 | WD repeat domain 13 [Source:HGNC Symbol;Acc:HGNC:14352] | 7695 | -0.134 | -0.2625 | No |
| 236 | H1-2 | "H1.2 linker histone, cluster member [Source:HGNC Symbol;Acc:HGNC:4716]" | 7710 | -0.135 | -0.2628 | No |
| 237 | HOXC13 | homeobox C13 [Source:HGNC Symbol;Acc:HGNC:5125] | 7738 | -0.137 | -0.2641 | No |
| 238 | BAHCC1 | BAH domain and coiled-coil containing 1 [Source:HGNC Symbol;Acc:HGNC:29279] | 7740 | -0.137 | -0.2633 | No |
| 239 | PWWP3A | "PWWP domain containing 3A, DNA repair factor [Source:HGNC Symbol;Acc:HGNC:29641]" | 7748 | -0.138 | -0.2629 | No |
| 240 | RBL1 | RB transcriptional corepressor like 1 [Source:HGNC Symbol;Acc:HGNC:9893] | 7776 | -0.140 | -0.2642 | No |
| 241 | SOX10 | SRY-box transcription factor 10 [Source:HGNC Symbol;Acc:HGNC:11190] | 7788 | -0.141 | -0.2642 | No |
| 242 | CHAF1A | chromatin assembly factor 1 subunit A [Source:HGNC Symbol;Acc:HGNC:1910] | 7836 | -0.143 | -0.2671 | No |
| 243 | MYOD1 | myogenic differentiation 1 [Source:HGNC Symbol;Acc:HGNC:7611] | 7839 | -0.143 | -0.2663 | No |
| 244 | GMNN | geminin DNA replication inhibitor [Source:HGNC Symbol;Acc:HGNC:17493] | 7866 | -0.146 | -0.2675 | No |
| 245 | HDAC1 | histone deacetylase 1 [Source:HGNC Symbol;Acc:HGNC:4852] | 7911 | -0.148 | -0.2700 | No |
| 246 | TLE4 | "TLE family member 4, transcriptional corepressor [Source:HGNC Symbol;Acc:HGNC:11840]" | 7915 | -0.149 | -0.2693 | No |
| 247 | NRL | neural retina leucine zipper [Source:HGNC Symbol;Acc:HGNC:8002] | 7936 | -0.150 | -0.2700 | No |
| 248 | ERCC6 | "ERCC excision repair 6, chromatin remodeling factor [Source:HGNC Symbol;Acc:HGNC:3438]" | 7939 | -0.150 | -0.2692 | No |
| 249 | FMR1 | FMRP translational regulator 1 [Source:HGNC Symbol;Acc:HGNC:3775] | 7944 | -0.151 | -0.2685 | No |
| 250 | ACTL6B | actin like 6B [Source:HGNC Symbol;Acc:HGNC:160] | 8002 | -0.155 | -0.2721 | No |
| 251 | SMARCC1 | "SWI/SNF related, matrix associated, actin dependent regulator of chromatin subfamily c member 1 [Source:HGNC Symbol;Acc:HGNC:11104]" | 8052 | -0.159 | -0.2750 | No |
| 252 | H1-3 | "H1.3 linker histone, cluster member [Source:HGNC Symbol;Acc:HGNC:4717]" | 8056 | -0.160 | -0.2743 | No |
| 253 | MCM3AP | minichromosome maintenance complex component 3 associated protein [Source:HGNC Symbol;Acc:HGNC:6946] | 8077 | -0.161 | -0.2748 | No |
| 254 | EXO1 | exonuclease 1 [Source:HGNC Symbol;Acc:HGNC:3511] | 8088 | -0.162 | -0.2746 | No |
| 255 | DHX30 | DExH-box helicase 30 [Source:HGNC Symbol;Acc:HGNC:16716] | 8111 | -0.164 | -0.2753 | No |
| 256 | BARX2 | BARX homeobox 2 [Source:HGNC Symbol;Acc:HGNC:956] | 8122 | -0.164 | -0.2751 | No |
| 257 | RING1 | ring finger protein 1 [Source:HGNC Symbol;Acc:HGNC:10018] | 8129 | -0.165 | -0.2745 | No |
| 258 | ZEB1 | zinc finger E-box binding homeobox 1 [Source:HGNC Symbol;Acc:HGNC:11642] | 8147 | -0.166 | -0.2748 | No |
| 259 | CCNT2 | cyclin T2 [Source:HGNC Symbol;Acc:HGNC:1600] | 8192 | -0.170 | -0.2772 | No |
| 260 | AR | androgen receptor [Source:HGNC Symbol;Acc:HGNC:644] | 8217 | -0.173 | -0.2780 | No |
| 261 | HOXD10 | homeobox D10 [Source:HGNC Symbol;Acc:HGNC:5133] | 8257 | -0.176 | -0.2801 | No |
| 262 | KDM3A | lysine demethylase 3A [Source:HGNC Symbol;Acc:HGNC:20815] | 8264 | -0.176 | -0.2794 | No |
| 263 | POU4F2 | POU class 4 homeobox 2 [Source:HGNC Symbol;Acc:HGNC:9219] | 8292 | -0.178 | -0.2804 | No |
| 264 | BRD3 | bromodomain containing 3 [Source:HGNC Symbol;Acc:HGNC:1104] | 8369 | -0.183 | -0.2854 | No |
| 265 | SVEP1 | "sushi, von Willebrand factor type A, EGF and pentraxin domain containing 1 [Source:HGNC Symbol;Acc:HGNC:15985]" | 8431 | -0.188 | -0.2891 | No |
| 266 | E2F4 | E2F transcription factor 4 [Source:HGNC Symbol;Acc:HGNC:3118] | 8445 | -0.190 | -0.2889 | No |
| 267 | STAT3 | signal transducer and activator of transcription 3 [Source:HGNC Symbol;Acc:HGNC:11364] | 8453 | -0.190 | -0.2882 | No |
| 268 | TASOR | transcription activation suppressor [Source:HGNC Symbol;Acc:HGNC:30314] | 8468 | -0.191 | -0.2881 | No |
| 269 | SMARCC2 | "SWI/SNF related, matrix associated, actin dependent regulator of chromatin subfamily c member 2 [Source:HGNC Symbol;Acc:HGNC:11105]" | 8480 | -0.192 | -0.2878 | No |
| 270 | LDB1 | LIM domain binding 1 [Source:HGNC Symbol;Acc:HGNC:6532] | 8522 | -0.195 | -0.2898 | No |
| 271 | SIRT6 | sirtuin 6 [Source:HGNC Symbol;Acc:HGNC:14934] | 8532 | -0.196 | -0.2893 | No |
| 272 | PRDM14 | PR/SET domain 14 [Source:HGNC Symbol;Acc:HGNC:14001] | 8553 | -0.198 | -0.2896 | No |
| 273 | PATZ1 | POZ/BTB and AT hook containing zinc finger 1 [Source:HGNC Symbol;Acc:HGNC:13071] | 8589 | -0.200 | -0.2912 | No |
| 274 | ATF2 | activating transcription factor 2 [Source:HGNC Symbol;Acc:HGNC:784] | 8592 | -0.201 | -0.2900 | No |
| 275 | KDM1A | lysine demethylase 1A [Source:HGNC Symbol;Acc:HGNC:29079] | 8608 | -0.202 | -0.2899 | No |
| 276 | ASXL1 | ASXL transcriptional regulator 1 [Source:HGNC Symbol;Acc:HGNC:18318] | 8610 | -0.202 | -0.2887 | No |
| 277 | ERCC3 | "ERCC excision repair 3, TFIIH core complex helicase subunit [Source:HGNC Symbol;Acc:HGNC:3435]" | 8669 | -0.206 | -0.2921 | No |
| 278 | MECP2 | methyl-CpG binding protein 2 [Source:HGNC Symbol;Acc:HGNC:6990] | 8687 | -0.208 | -0.2921 | No |
| 279 | SSRP1 | structure specific recognition protein 1 [Source:HGNC Symbol;Acc:HGNC:11327] | 8692 | -0.208 | -0.2911 | No |
| 280 | RARG | retinoic acid receptor gamma [Source:HGNC Symbol;Acc:HGNC:9866] | 8700 | -0.209 | -0.2903 | No |
| 281 | SIRT7 | sirtuin 7 [Source:HGNC Symbol;Acc:HGNC:14935] | 8733 | -0.211 | -0.2915 | No |
| 282 | RERE | arginine-glutamic acid dipeptide repeats [Source:HGNC Symbol;Acc:HGNC:9965] | 8744 | -0.212 | -0.2910 | No |
| 283 | BAHD1 | bromo adjacent homology domain containing 1 [Source:HGNC Symbol;Acc:HGNC:29153] | 8745 | -0.212 | -0.2896 | No |
| 284 | SUPT6H | "SPT6 homolog, histone chaperone and transcription elongation factor [Source:HGNC Symbol;Acc:HGNC:11470]" | 8752 | -0.213 | -0.2887 | No |
| 285 | APEX1 | apurinic/apyrimidinic endodeoxyribonuclease 1 [Source:HGNC Symbol;Acc:HGNC:587] | 8767 | -0.213 | -0.2885 | No |
| 286 | MCM9 | minichromosome maintenance 9 homologous recombination repair factor [Source:HGNC Symbol;Acc:HGNC:21484] | 8781 | -0.215 | -0.2881 | No |
| 287 | RBPJ | recombination signal binding protein for immunoglobulin kappa J region [Source:HGNC Symbol;Acc:HGNC:5724] | 8800 | -0.217 | -0.2882 | No |
| 288 | MSH6 | mutS homolog 6 [Source:HGNC Symbol;Acc:HGNC:7329] | 8846 | -0.221 | -0.2904 | No |
| 289 | PRKAA2 | protein kinase AMP-activated catalytic subunit alpha 2 [Source:HGNC Symbol;Acc:HGNC:9377] | 8864 | -0.222 | -0.2903 | No |
| 290 | PBRM1 | polybromo 1 [Source:HGNC Symbol;Acc:HGNC:30064] | 8887 | -0.224 | -0.2907 | No |
| 291 | GTF2F1 | general transcription factor IIF subunit 1 [Source:HGNC Symbol;Acc:HGNC:4652] | 8910 | -0.225 | -0.2910 | No |
| 292 | ZNF276 | zinc finger protein 276 [Source:HGNC Symbol;Acc:HGNC:23330] | 8915 | -0.226 | -0.2899 | No |
| 293 | CHD4 | chromodomain helicase DNA binding protein 4 [Source:HGNC Symbol;Acc:HGNC:1919] | 8917 | -0.226 | -0.2885 | No |
| 294 | RBMX | RNA binding motif protein X-linked [Source:HGNC Symbol;Acc:HGNC:9910] | 8938 | -0.227 | -0.2886 | No |
| 295 | SIRT1 | sirtuin 1 [Source:HGNC Symbol;Acc:HGNC:14929] | 8947 | -0.227 | -0.2878 | No |
| 296 | NCAPH2 | non-SMC condensin II complex subunit H2 [Source:HGNC Symbol;Acc:HGNC:25071] | 8983 | -0.230 | -0.2892 | No |
| 297 | SPI1 | Spi-1 proto-oncogene [Source:HGNC Symbol;Acc:HGNC:11241] | 9012 | -0.232 | -0.2899 | No |
| 298 | ZC3H12A | zinc finger CCCH-type containing 12A [Source:HGNC Symbol;Acc:HGNC:26259] | 9030 | -0.234 | -0.2898 | No |
| 299 | SMAD4 | SMAD family member 4 [Source:HGNC Symbol;Acc:HGNC:6770] | 9035 | -0.235 | -0.2886 | No |
| 300 | RNF2 | ring finger protein 2 [Source:HGNC Symbol;Acc:HGNC:10061] | 9038 | -0.235 | -0.2872 | No |
| 301 | HP1BP3 | heterochromatin protein 1 binding protein 3 [Source:HGNC Symbol;Acc:HGNC:24973] | 9044 | -0.235 | -0.2861 | No |
| 302 | TDRD3 | tudor domain containing 3 [Source:HGNC Symbol;Acc:HGNC:20612] | 9080 | -0.238 | -0.2874 | No |
| 303 | RCOR1 | REST corepressor 1 [Source:HGNC Symbol;Acc:HGNC:17441] | 9120 | -0.241 | -0.2890 | No |
| 304 | DLX2 | distal-less homeobox 2 [Source:HGNC Symbol;Acc:HGNC:2915] | 9168 | -0.245 | -0.2912 | No |
| 305 | ZNF143 | zinc finger protein 143 [Source:HGNC Symbol;Acc:HGNC:12928] | 9246 | -0.252 | -0.2958 | No |
| 306 | NCOA1 | nuclear receptor coactivator 1 [Source:HGNC Symbol;Acc:HGNC:7668] | 9270 | -0.253 | -0.2960 | No |
| 307 | POU4F1 | POU class 4 homeobox 1 [Source:HGNC Symbol;Acc:HGNC:9218] | 9300 | -0.256 | -0.2967 | No |
| 308 | EP400 | E1A binding protein p400 [Source:HGNC Symbol;Acc:HGNC:11958] | 9304 | -0.256 | -0.2953 | No |
| 309 | CENPA | centromere protein A [Source:HGNC Symbol;Acc:HGNC:1851] | 9311 | -0.256 | -0.2941 | No |
| 310 | NONO | non-POU domain containing octamer binding [Source:HGNC Symbol;Acc:HGNC:7871] | 9334 | -0.258 | -0.2942 | No |
| 311 | HDAC2 | histone deacetylase 2 [Source:HGNC Symbol;Acc:HGNC:4853] | 9359 | -0.260 | -0.2945 | No |
| 312 | GTF2H1 | general transcription factor IIH subunit 1 [Source:HGNC Symbol;Acc:HGNC:4655] | 9455 | -0.269 | -0.3004 | No |
| 313 | RAN | "RAN, member RAS oncogene family [Source:HGNC Symbol;Acc:HGNC:9846]" | 9478 | -0.272 | -0.3004 | No |
| 314 | PRDM13 | PR/SET domain 13 [Source:HGNC Symbol;Acc:HGNC:13998] | 9484 | -0.273 | -0.2991 | No |
| 315 | NELFA | negative elongation factor complex member A [Source:HGNC Symbol;Acc:HGNC:12768] | 9488 | -0.273 | -0.2976 | No |
| 316 | TSPYL2 | TSPY like 2 [Source:HGNC Symbol;Acc:HGNC:24358] | 9491 | -0.273 | -0.2960 | No |
| 317 | H3-3A | H3.3 histone A [Source:HGNC Symbol;Acc:HGNC:4764] | 9503 | -0.274 | -0.2951 | No |
| 318 | RAD21L1 | RAD21 cohesin complex component like 1 [Source:HGNC Symbol;Acc:HGNC:16271] | 9510 | -0.275 | -0.2938 | No |
| 319 | TSPY2 | testis specific protein Y-linked 2 [Source:HGNC Symbol;Acc:HGNC:23924] | 9518 | -0.276 | -0.2926 | No |
| 320 | ERG | ETS transcription factor ERG [Source:HGNC Symbol;Acc:HGNC:3446] | 9543 | -0.278 | -0.2928 | No |
| 321 | YBX1 | Y-box binding protein 1 [Source:HGNC Symbol;Acc:HGNC:8014] | 9623 | -0.285 | -0.2973 | No |
| 322 | SNAI2 | snail family transcriptional repressor 2 [Source:HGNC Symbol;Acc:HGNC:11094] | 9697 | -0.291 | -0.3013 | No |
| 323 | SFPQ | splicing factor proline and glutamine rich [Source:HGNC Symbol;Acc:HGNC:10774] | 9747 | -0.296 | -0.3033 | No |
| 324 | MPO | myeloperoxidase [Source:HGNC Symbol;Acc:HGNC:7218] | 9792 | -0.301 | -0.3049 | Yes |
| 325 | KAT2A | lysine acetyltransferase 2A [Source:HGNC Symbol;Acc:HGNC:4201] | 9799 | -0.301 | -0.3035 | Yes |
| 326 | PBX2 | PBX homeobox 2 [Source:HGNC Symbol;Acc:HGNC:8633] | 9804 | -0.302 | -0.3019 | Yes |
| 327 | THRB | thyroid hormone receptor beta [Source:HGNC Symbol;Acc:HGNC:11799] | 9820 | -0.303 | -0.3011 | Yes |
| 328 | TOX4 | TOX high mobility group box family member 4 [Source:HGNC Symbol;Acc:HGNC:20161] | 9823 | -0.303 | -0.2993 | Yes |
| 329 | HNRNPU | heterogeneous nuclear ribonucleoprotein U [Source:HGNC Symbol;Acc:HGNC:5048] | 9847 | -0.306 | -0.2992 | Yes |
| 330 | NEUROG1 | neurogenin 1 [Source:HGNC Symbol;Acc:HGNC:7764] | 9856 | -0.307 | -0.2979 | Yes |
| 331 | NKX2-5 | NK2 homeobox 5 [Source:HGNC Symbol;Acc:HGNC:2488] | 9875 | -0.309 | -0.2974 | Yes |
| 332 | SATB2 | SATB homeobox 2 [Source:HGNC Symbol;Acc:HGNC:21637] | 9883 | -0.310 | -0.2959 | Yes |
| 333 | TRIM24 | tripartite motif containing 24 [Source:HGNC Symbol;Acc:HGNC:11812] | 9927 | -0.314 | -0.2974 | Yes |
| 334 | CORT | cortistatin [Source:HGNC Symbol;Acc:HGNC:2257] | 9964 | -0.317 | -0.2982 | Yes |
| 335 | HMGA1 | high mobility group AT-hook 1 [Source:HGNC Symbol;Acc:HGNC:5010] | 9966 | -0.318 | -0.2963 | Yes |
| 336 | ZKSCAN3 | zinc finger with KRAB and SCAN domains 3 [Source:HGNC Symbol;Acc:HGNC:13853] | 9970 | -0.318 | -0.2945 | Yes |
| 337 | CDC45 | cell division cycle 45 [Source:HGNC Symbol;Acc:HGNC:1739] | 9982 | -0.319 | -0.2933 | Yes |
| 338 | SMARCA4 | "SWI/SNF related, matrix associated, actin dependent regulator of chromatin, subfamily a, member 4 [Source:HGNC Symbol;Acc:HGNC:11100]" | 10009 | -0.322 | -0.2933 | Yes |
| 339 | WRN | WRN RecQ like helicase [Source:HGNC Symbol;Acc:HGNC:12791] | 10010 | -0.323 | -0.2912 | Yes |
| 340 | POLR3G | RNA polymerase III subunit G [Source:HGNC Symbol;Acc:HGNC:30075] | 10018 | -0.323 | -0.2897 | Yes |
| 341 | CBX2 | chromobox 2 [Source:HGNC Symbol;Acc:HGNC:1552] | 10065 | -0.327 | -0.2913 | Yes |
| 342 | DNAJC2 | DnaJ heat shock protein family (Hsp40) member C2 [Source:HGNC Symbol;Acc:HGNC:13192] | 10081 | -0.329 | -0.2904 | Yes |
| 343 | SFMBT1 | Scm like with four mbt domains 1 [Source:HGNC Symbol;Acc:HGNC:20255] | 10094 | -0.330 | -0.2893 | Yes |
| 344 | SUZ12 | SUZ12 polycomb repressive complex 2 subunit [Source:HGNC Symbol;Acc:HGNC:17101] | 10104 | -0.331 | -0.2879 | Yes |
| 345 | IKZF5 | IKAROS family zinc finger 5 [Source:HGNC Symbol;Acc:HGNC:14283] | 10109 | -0.331 | -0.2861 | Yes |
| 346 | SMARCB1 | "SWI/SNF related, matrix associated, actin dependent regulator of chromatin, subfamily b, member 1 [Source:HGNC Symbol;Acc:HGNC:11103]" | 10117 | -0.333 | -0.2845 | Yes |
| 347 | RARA | retinoic acid receptor alpha [Source:HGNC Symbol;Acc:HGNC:9864] | 10141 | -0.335 | -0.2842 | Yes |
| 348 | BAP1 | BRCA1 associated protein 1 [Source:HGNC Symbol;Acc:HGNC:950] | 10151 | -0.336 | -0.2827 | Yes |
| 349 | TTF1 | transcription termination factor 1 [Source:HGNC Symbol;Acc:HGNC:12397] | 10161 | -0.337 | -0.2813 | Yes |
| 350 | CTBP1 | C-terminal binding protein 1 [Source:HGNC Symbol;Acc:HGNC:2494] | 10188 | -0.339 | -0.2812 | Yes |
| 351 | REPIN1 | replication initiator 1 [Source:HGNC Symbol;Acc:HGNC:17922] | 10195 | -0.340 | -0.2795 | Yes |
| 352 | NR5A1 | nuclear receptor subfamily 5 group A member 1 [Source:HGNC Symbol;Acc:HGNC:7983] | 10308 | -0.351 | -0.2863 | Yes |
| 353 | PRDM1 | PR/SET domain 1 [Source:HGNC Symbol;Acc:HGNC:9346] | 10374 | -0.358 | -0.2892 | Yes |
| 354 | PDX1 | pancreatic and duodenal homeobox 1 [Source:HGNC Symbol;Acc:HGNC:6107] | 10409 | -0.362 | -0.2896 | Yes |
| 355 | ACTN4 | actinin alpha 4 [Source:HGNC Symbol;Acc:HGNC:166] | 10472 | -0.370 | -0.2922 | Yes |
| 356 | PHC1 | polyhomeotic homolog 1 [Source:HGNC Symbol;Acc:HGNC:3182] | 10486 | -0.372 | -0.2909 | Yes |
| 357 | PPARGC1A | PPARG coactivator 1 alpha [Source:HGNC Symbol;Acc:HGNC:9237] | 10504 | -0.374 | -0.2898 | Yes |
| 358 | RAD21 | RAD21 cohesin complex component [Source:HGNC Symbol;Acc:HGNC:9811] | 10510 | -0.374 | -0.2878 | Yes |
| 359 | WAC | WW domain containing adaptor with coiled-coil [Source:HGNC Symbol;Acc:HGNC:17327] | 10514 | -0.375 | -0.2857 | Yes |
| 360 | MTA2 | metastasis associated 1 family member 2 [Source:HGNC Symbol;Acc:HGNC:7411] | 10600 | -0.383 | -0.2900 | Yes |
| 361 | DHX9 | DExH-box helicase 9 [Source:HGNC Symbol;Acc:HGNC:2750] | 10627 | -0.386 | -0.2896 | Yes |
| 362 | FOS | "Fos proto-oncogene, AP-1 transcription factor subunit [Source:HGNC Symbol;Acc:HGNC:3796]" | 10640 | -0.387 | -0.2881 | Yes |
| 363 | TFAP2B | transcription factor AP-2 beta [Source:HGNC Symbol;Acc:HGNC:11743] | 10643 | -0.387 | -0.2858 | Yes |
| 364 | MLLT10 | MLLT10 histone lysine methyltransferase DOT1L cofactor [Source:HGNC Symbol;Acc:HGNC:16063] | 10645 | -0.387 | -0.2834 | Yes |
| 365 | RBPJL | recombination signal binding protein for immunoglobulin kappa J region like [Source:HGNC Symbol;Acc:HGNC:13761] | 10650 | -0.388 | -0.2812 | Yes |
| 366 | CKS2 | CDC28 protein kinase regulatory subunit 2 [Source:HGNC Symbol;Acc:HGNC:2000] | 10695 | -0.392 | -0.2822 | Yes |
| 367 | FAAP24 | FA core complex associated protein 24 [Source:HGNC Symbol;Acc:HGNC:28467] | 10723 | -0.396 | -0.2819 | Yes |
| 368 | GLYR1 | glyoxylate reductase 1 homolog [Source:HGNC Symbol;Acc:HGNC:24434] | 10807 | -0.406 | -0.2859 | Yes |
| 369 | ACTL6A | actin like 6A [Source:HGNC Symbol;Acc:HGNC:24124] | 10821 | -0.407 | -0.2844 | Yes |
| 370 | WDR82 | WD repeat domain 82 [Source:HGNC Symbol;Acc:HGNC:28826] | 10854 | -0.411 | -0.2843 | Yes |
| 371 | ELK4 | ETS transcription factor ELK4 [Source:HGNC Symbol;Acc:HGNC:3326] | 10862 | -0.411 | -0.2822 | Yes |
| 372 | NUP98 | nucleoporin 98 and 96 precursor [Source:HGNC Symbol;Acc:HGNC:8068] | 10875 | -0.413 | -0.2805 | Yes |
| 373 | IRF3 | interferon regulatory factor 3 [Source:HGNC Symbol;Acc:HGNC:6118] | 10879 | -0.414 | -0.2781 | Yes |
| 374 | CREBBP | CREB binding protein [Source:HGNC Symbol;Acc:HGNC:2348] | 10881 | -0.414 | -0.2755 | Yes |
| 375 | SBNO1 | strawberry notch homolog 1 [Source:HGNC Symbol;Acc:HGNC:22973] | 10924 | -0.421 | -0.2762 | Yes |
| 376 | MNT | MAX network transcriptional repressor [Source:HGNC Symbol;Acc:HGNC:7188] | 10952 | -0.424 | -0.2756 | Yes |
| 377 | GATA1 | GATA binding protein 1 [Source:HGNC Symbol;Acc:HGNC:4170] | 10958 | -0.425 | -0.2733 | Yes |
| 378 | REC8 | REC8 meiotic recombination protein [Source:HGNC Symbol;Acc:HGNC:16879] | 11016 | -0.432 | -0.2751 | Yes |
| 379 | POLE | "DNA polymerase epsilon, catalytic subunit [Source:HGNC Symbol;Acc:HGNC:9177]" | 11026 | -0.433 | -0.2731 | Yes |
| 380 | GATA2 | GATA binding protein 2 [Source:HGNC Symbol;Acc:HGNC:4171] | 11058 | -0.439 | -0.2727 | Yes |
| 381 | CDK9 | cyclin dependent kinase 9 [Source:HGNC Symbol;Acc:HGNC:1780] | 11071 | -0.441 | -0.2709 | Yes |
| 382 | KDM5A | lysine demethylase 5A [Source:HGNC Symbol;Acc:HGNC:9886] | 11097 | -0.443 | -0.2700 | Yes |
| 383 | CBX3 | chromobox 3 [Source:HGNC Symbol;Acc:HGNC:1553] | 11113 | -0.445 | -0.2684 | Yes |
| 384 | MED1 | mediator complex subunit 1 [Source:HGNC Symbol;Acc:HGNC:9234] | 11146 | -0.450 | -0.2681 | Yes |
| 385 | HSF1 | heat shock transcription factor 1 [Source:HGNC Symbol;Acc:HGNC:5224] | 11189 | -0.456 | -0.2685 | Yes |
| 386 | GLI2 | GLI family zinc finger 2 [Source:HGNC Symbol;Acc:HGNC:4318] | 11195 | -0.456 | -0.2660 | Yes |
| 387 | SKIL | SKI like proto-oncogene [Source:HGNC Symbol;Acc:HGNC:10897] | 11253 | -0.464 | -0.2676 | Yes |
| 388 | TNRC18 | trinucleotide repeat containing 18 [Source:HGNC Symbol;Acc:HGNC:11962] | 11279 | -0.468 | -0.2666 | Yes |
| 389 | FOXO3 | forkhead box O3 [Source:HGNC Symbol;Acc:HGNC:3821] | 11305 | -0.472 | -0.2656 | Yes |
| 390 | NKX6-1 | NK6 homeobox 1 [Source:HGNC Symbol;Acc:HGNC:7839] | 11314 | -0.473 | -0.2632 | Yes |
| 391 | NR5A2 | nuclear receptor subfamily 5 group A member 2 [Source:HGNC Symbol;Acc:HGNC:7984] | 11362 | -0.481 | -0.2639 | Yes |
| 392 | NOC3L | NOC3 like DNA replication regulator [Source:HGNC Symbol;Acc:HGNC:24034] | 11365 | -0.481 | -0.2609 | Yes |
| 393 | ZFX | zinc finger protein X-linked [Source:HGNC Symbol;Acc:HGNC:12869] | 11372 | -0.482 | -0.2583 | Yes |
| 394 | PROP1 | PROP paired-like homeobox 1 [Source:HGNC Symbol;Acc:HGNC:9455] | 11373 | -0.482 | -0.2552 | Yes |
| 395 | PRKAA1 | protein kinase AMP-activated catalytic subunit alpha 1 [Source:HGNC Symbol;Acc:HGNC:9376] | 11499 | -0.501 | -0.2621 | Yes |
| 396 | MORC2 | MORC family CW-type zinc finger 2 [Source:HGNC Symbol;Acc:HGNC:23573] | 11523 | -0.504 | -0.2607 | Yes |
| 397 | SCML2 | Scm polycomb group protein like 2 [Source:HGNC Symbol;Acc:HGNC:10581] | 11567 | -0.510 | -0.2609 | Yes |
| 398 | PHF8 | PHD finger protein 8 [Source:HGNC Symbol;Acc:HGNC:20672] | 11592 | -0.514 | -0.2595 | Yes |
| 399 | BMI1 | "BMI1 proto-oncogene, polycomb ring finger [Source:HGNC Symbol;Acc:HGNC:1066]" | 11639 | -0.523 | -0.2598 | Yes |
| 400 | NAP1L1 | nucleosome assembly protein 1 like 1 [Source:HGNC Symbol;Acc:HGNC:7637] | 11685 | -0.530 | -0.2600 | Yes |
| 401 | GRWD1 | glutamate rich WD repeat containing 1 [Source:HGNC Symbol;Acc:HGNC:21270] | 11694 | -0.531 | -0.2573 | Yes |
| 402 | PHC2 | polyhomeotic homolog 2 [Source:HGNC Symbol;Acc:HGNC:3183] | 11701 | -0.533 | -0.2543 | Yes |
| 403 | TTC21B | tetratricopeptide repeat domain 21B [Source:HGNC Symbol;Acc:HGNC:25660] | 11713 | -0.535 | -0.2518 | Yes |
| 404 | SUPT5H | "SPT5 homolog, DSIF elongation factor subunit [Source:HGNC Symbol;Acc:HGNC:11469]" | 11735 | -0.539 | -0.2500 | Yes |
| 405 | EP300 | E1A binding protein p300 [Source:HGNC Symbol;Acc:HGNC:3373] | 11741 | -0.540 | -0.2469 | Yes |
| 406 | CHD8 | chromodomain helicase DNA binding protein 8 [Source:HGNC Symbol;Acc:HGNC:20153] | 11825 | -0.554 | -0.2500 | Yes |
| 407 | TOP1 | DNA topoisomerase I [Source:HGNC Symbol;Acc:HGNC:11986] | 11828 | -0.555 | -0.2466 | Yes |
| 408 | KDM6B | lysine demethylase 6B [Source:HGNC Symbol;Acc:HGNC:29012] | 11835 | -0.556 | -0.2436 | Yes |
| 409 | SMARCA5 | "SWI/SNF related, matrix associated, actin dependent regulator of chromatin, subfamily a, member 5 [Source:HGNC Symbol;Acc:HGNC:11101]" | 11838 | -0.557 | -0.2401 | Yes |
| 410 | SBNO2 | strawberry notch homolog 2 [Source:HGNC Symbol;Acc:HGNC:29158] | 11850 | -0.560 | -0.2374 | Yes |
| 411 | STAG3L1 | stromal antigen 3-like 1 (pseudogene) [Source:HGNC Symbol;Acc:HGNC:33852] | 11874 | -0.566 | -0.2356 | Yes |
| 412 | PHC3 | polyhomeotic homolog 3 [Source:HGNC Symbol;Acc:HGNC:15682] | 11881 | -0.568 | -0.2325 | Yes |
| 413 | SCML1 | Scm polycomb group protein like 1 [Source:HGNC Symbol;Acc:HGNC:10580] | 11890 | -0.570 | -0.2294 | Yes |
| 414 | NUCKS1 | nuclear casein kinase and cyclin dependent kinase substrate 1 [Source:HGNC Symbol;Acc:HGNC:29923] | 11945 | -0.581 | -0.2301 | Yes |
| 415 | UTY | "ubiquitously transcribed tetratricopeptide repeat containing, Y-linked [Source:HGNC Symbol;Acc:HGNC:12638]" | 11984 | -0.590 | -0.2293 | Yes |
| 416 | SMARCD1 | "SWI/SNF related, matrix associated, actin dependent regulator of chromatin, subfamily d, member 1 [Source:HGNC Symbol;Acc:HGNC:11106]" | 11991 | -0.592 | -0.2260 | Yes |
| 417 | ATF4 | activating transcription factor 4 [Source:HGNC Symbol;Acc:HGNC:786] | 11999 | -0.593 | -0.2228 | Yes |
| 418 | SET | SET nuclear proto-oncogene [Source:HGNC Symbol;Acc:HGNC:10760] | 12000 | -0.593 | -0.2189 | Yes |
| 419 | JARID2 | jumonji and AT-rich interaction domain containing 2 [Source:HGNC Symbol;Acc:HGNC:6196] | 12030 | -0.600 | -0.2174 | Yes |
| 420 | EGR1 | early growth response 1 [Source:HGNC Symbol;Acc:HGNC:3238] | 12052 | -0.607 | -0.2152 | Yes |
| 421 | EGFR | epidermal growth factor receptor [Source:HGNC Symbol;Acc:HGNC:3236] | 12109 | -0.622 | -0.2157 | Yes |
| 422 | VCX | variable charge X-linked [Source:HGNC Symbol;Acc:HGNC:12667] | 12110 | -0.623 | -0.2117 | Yes |
| 423 | GABPA | GA binding protein transcription factor subunit alpha [Source:HGNC Symbol;Acc:HGNC:4071] | 12117 | -0.624 | -0.2082 | Yes |
| 424 | HIRA | histone cell cycle regulator [Source:HGNC Symbol;Acc:HGNC:4916] | 12143 | -0.630 | -0.2061 | Yes |
| 425 | TFAM | "transcription factor A, mitochondrial [Source:HGNC Symbol;Acc:HGNC:11741]" | 12175 | -0.637 | -0.2045 | Yes |
| 426 | MBTD1 | mbt domain containing 1 [Source:HGNC Symbol;Acc:HGNC:19866] | 12189 | -0.641 | -0.2015 | Yes |
| 427 | TADA2A | transcriptional adaptor 2A [Source:HGNC Symbol;Acc:HGNC:11531] | 12217 | -0.650 | -0.1995 | Yes |
| 428 | FOXC2 | forkhead box C2 [Source:HGNC Symbol;Acc:HGNC:3801] | 12218 | -0.650 | -0.1953 | Yes |
| 429 | YY1 | YY1 transcription factor [Source:HGNC Symbol;Acc:HGNC:12856] | 12229 | -0.653 | -0.1919 | Yes |
| 430 | FOXO4 | forkhead box O4 [Source:HGNC Symbol;Acc:HGNC:7139] | 12240 | -0.656 | -0.1885 | Yes |
| 431 | MCMBP | minichromosome maintenance complex binding protein [Source:HGNC Symbol;Acc:HGNC:25782] | 12243 | -0.656 | -0.1844 | Yes |
| 432 | NOC2L | NOC2 like nucleolar associated transcriptional repressor [Source:HGNC Symbol;Acc:HGNC:24517] | 12258 | -0.663 | -0.1813 | Yes |
| 433 | UBTF | upstream binding transcription factor [Source:HGNC Symbol;Acc:HGNC:12511] | 12261 | -0.665 | -0.1772 | Yes |
| 434 | NFIA | nuclear factor I A [Source:HGNC Symbol;Acc:HGNC:7784] | 12294 | -0.675 | -0.1754 | Yes |
| 435 | ATF5 | activating transcription factor 5 [Source:HGNC Symbol;Acc:HGNC:790] | 12310 | -0.680 | -0.1723 | Yes |
| 436 | MYOG | myogenin [Source:HGNC Symbol;Acc:HGNC:7612] | 12313 | -0.681 | -0.1680 | Yes |
| 437 | TAF2 | TATA-box binding protein associated factor 2 [Source:HGNC Symbol;Acc:HGNC:11536] | 12328 | -0.684 | -0.1648 | Yes |
| 438 | GLI1 | GLI family zinc finger 1 [Source:HGNC Symbol;Acc:HGNC:4317] | 12333 | -0.687 | -0.1607 | Yes |
| 439 | POLG | "DNA polymerase gamma, catalytic subunit [Source:HGNC Symbol;Acc:HGNC:9179]" | 12369 | -0.699 | -0.1590 | Yes |
| 440 | REST | RE1 silencing transcription factor [Source:HGNC Symbol;Acc:HGNC:9966] | 12382 | -0.703 | -0.1554 | Yes |
| 441 | NIPBL | NIPBL cohesin loading factor [Source:HGNC Symbol;Acc:HGNC:28862] | 12385 | -0.703 | -0.1511 | Yes |
| 442 | SAFB | scaffold attachment factor B [Source:HGNC Symbol;Acc:HGNC:10520] | 12395 | -0.705 | -0.1473 | Yes |
| 443 | ASH1L | ASH1 like histone lysine methyltransferase [Source:HGNC Symbol;Acc:HGNC:19088] | 12405 | -0.708 | -0.1434 | Yes |
| 444 | POLR3D | RNA polymerase III subunit D [Source:HGNC Symbol;Acc:HGNC:1080] | 12490 | -0.744 | -0.1454 | Yes |
| 445 | CITED1 | Cbp/p300 interacting transactivator with Glu/Asp rich carboxy-terminal domain 1 [Source:HGNC Symbol;Acc:HGNC:1986] | 12493 | -0.746 | -0.1408 | Yes |
| 446 | PER1 | period circadian regulator 1 [Source:HGNC Symbol;Acc:HGNC:8845] | 12496 | -0.746 | -0.1362 | Yes |
| 447 | FOSL2 | "FOS like 2, AP-1 transcription factor subunit [Source:HGNC Symbol;Acc:HGNC:3798]" | 12510 | -0.751 | -0.1324 | Yes |
| 448 | CCNT1 | cyclin T1 [Source:HGNC Symbol;Acc:HGNC:1599] | 12517 | -0.757 | -0.1280 | Yes |
| 449 | NPM1 | nucleophosmin 1 [Source:HGNC Symbol;Acc:HGNC:7910] | 12530 | -0.762 | -0.1241 | Yes |
| 450 | SRF | serum response factor [Source:HGNC Symbol;Acc:HGNC:11291] | 12549 | -0.773 | -0.1205 | Yes |
| 451 | NOTCH1 | notch receptor 1 [Source:HGNC Symbol;Acc:HGNC:7881] | 12555 | -0.776 | -0.1160 | Yes |
| 452 | CHD7 | chromodomain helicase DNA binding protein 7 [Source:HGNC Symbol;Acc:HGNC:20626] | 12591 | -0.791 | -0.1137 | Yes |
| 453 | PHF21A | PHD finger protein 21A [Source:HGNC Symbol;Acc:HGNC:24156] | 12594 | -0.792 | -0.1088 | Yes |
| 454 | BRD4 | bromodomain containing 4 [Source:HGNC Symbol;Acc:HGNC:13575] | 12606 | -0.800 | -0.1045 | Yes |
| 455 | DDX11 | DEAD/H-box helicase 11 [Source:HGNC Symbol;Acc:HGNC:2736] | 12607 | -0.803 | -0.0993 | Yes |
| 456 | AIRE | autoimmune regulator [Source:HGNC Symbol;Acc:HGNC:360] | 12610 | -0.804 | -0.0943 | Yes |
| 457 | PELP1 | "proline, glutamate and leucine rich protein 1 [Source:HGNC Symbol;Acc:HGNC:30134]" | 12634 | -0.814 | -0.0910 | Yes |
| 458 | ZNF609 | zinc finger protein 609 [Source:HGNC Symbol;Acc:HGNC:29003] | 12635 | -0.814 | -0.0857 | Yes |
| 459 | BCL6 | BCL6 transcription repressor [Source:HGNC Symbol;Acc:HGNC:1001] | 12667 | -0.838 | -0.0828 | Yes |
| 460 | HES1 | hes family bHLH transcription factor 1 [Source:HGNC Symbol;Acc:HGNC:5192] | 12676 | -0.849 | -0.0780 | Yes |
| 461 | RCC1 | regulator of chromosome condensation 1 [Source:HGNC Symbol;Acc:HGNC:1913] | 12699 | -0.864 | -0.0742 | Yes |
| 462 | ARID5A | AT-rich interaction domain 5A [Source:HGNC Symbol;Acc:HGNC:17361] | 12705 | -0.867 | -0.0691 | Yes |
| 463 | BRD2 | bromodomain containing 2 [Source:HGNC Symbol;Acc:HGNC:1103] | 12709 | -0.871 | -0.0637 | Yes |
| 464 | FUS | FUS RNA binding protein [Source:HGNC Symbol;Acc:HGNC:4010] | 12739 | -0.902 | -0.0602 | Yes |
| 465 | ACTB | actin beta [Source:HGNC Symbol;Acc:HGNC:132] | 12757 | -0.920 | -0.0557 | Yes |
| 466 | MTA1 | metastasis associated 1 [Source:HGNC Symbol;Acc:HGNC:7410] | 12792 | -0.952 | -0.0523 | Yes |
| 467 | CEBPB | CCAAT enhancer binding protein beta [Source:HGNC Symbol;Acc:HGNC:1834] | 12804 | -0.973 | -0.0469 | Yes |
| 468 | SIN3B | SIN3 transcription regulator family member B [Source:HGNC Symbol;Acc:HGNC:19354] | 12809 | -0.984 | -0.0409 | Yes |
| 469 | APBB1 | amyloid beta precursor protein binding family B member 1 [Source:HGNC Symbol;Acc:HGNC:581] | 12811 | -0.992 | -0.0346 | Yes |
| 470 | JUN | "Jun proto-oncogene, AP-1 transcription factor subunit [Source:HGNC Symbol;Acc:HGNC:6204]" | 12813 | -0.994 | -0.0283 | Yes |
| 471 | DDX5 | DEAD-box helicase 5 [Source:HGNC Symbol;Acc:HGNC:2746] | 12834 | -1.038 | -0.0233 | Yes |
| 472 | KLF4 | Kruppel like factor 4 [Source:HGNC Symbol;Acc:HGNC:6348] | 12848 | -1.058 | -0.0175 | Yes |
| 473 | EBF2 | EBF transcription factor 2 [Source:HGNC Symbol;Acc:HGNC:19090] | 12859 | -1.088 | -0.0113 | Yes |
| 474 | CTNNB1 | catenin beta 1 [Source:HGNC Symbol;Acc:HGNC:2514] | 12866 | -1.119 | -0.0046 | Yes |
| 475 | HDAC7 | histone deacetylase 7 [Source:HGNC Symbol;Acc:HGNC:14067] | 12870 | -1.131 | 0.0024 | Yes |
Table: GSEA details [plain text format]

  

Fig 2: GOMF\_CHROMATIN\_BINDING      
 Blue-Pink O' Gram in the Space of the Analyzed GeneSet

  

Fig 3: GOMF\_CHROMATIN\_BINDING: Random ES distribution      
 Gene set null distribution of ES for **GOMF\_CHROMATIN\_BINDING**
